# The three-dimensional morphology of mandible and glenoid fossa as contributing factors to menton deviation in facial asymmetry—retrospective study

**DOI:** 10.1186/s40510-020-00335-3

**Published:** 2020-09-22

**Authors:** Min-Hee Oh, Jin-Hyoung Cho

**Affiliations:** Department of Orthodontics, School of Dentistry, Dental 4D Research Institute, Dental Science Research Institute, Chonnam National University, 77 Yongbong-ro, Buk-gu, Gwangju, 61186 Korea

**Keywords:** Facial asymmetry, Menton deviation, 3D morphology, Computed tomography

## Abstract

**Background:**

The aim of this study is to evaluate whether the three-dimensional (3D) morphology of the mandibular condyle, glenoid fossa, and mandible correlated with menton deviation in facial asymmetry.

**Subjects and methods:**

Thirty adults (15 males and 15 females; mean age, 23.2 ± 3.8 years) with facial asymmetry were included. Linear, angular, and volumetric measurements of the 3D morphology of the mandibular condyle, glenoid fossa, and mandible were recorded using computed tomography (CT) images. The right/left differences were obtained by subtracting the left value from the right value, and an independent *t* test was used to compare the differences between the females and males. Multiple regression analysis was performed to identify the correlation between the right/left difference of the 3D morphology and menton deviation.

**Results:**

The results of the comparative analysis did not show any statistical difference between the females and males (*P* > .05), so the females and males were combined. Multiple regression analysis for the mandibular condyle, glenoid fossa, and mandible showed that neck length, ramus length, and frontal ramal inclination had positive influences on menton deviation, with 76.5% of explanatory power. The neck length and head volume of the mandibular condyle when only the mandibular condyle was considered, and the ramus length and frontal ramal inclination when only the mandible was considered had positive influence on menton deviation with 69.9% and 68.6% explanatory power, respectively. On the other hand, when only considering glenoid fossa, the glenoid fossa had little effect on menton deviation with 15.7% of explanatory power.

**Conclusions:**

In facial asymmetry, the right/left differences in mandibular condyle and mandible have more impact on the menton deviation than the right/left differences in glenoid fossa.

**Trial registration:**

CNUDH, CNUDH-EXP-2017-016. Registered 28 September 2017

## Background

Facial asymmetry is influenced more by the lower third of the face than by the upper and middle thirds of the face [1, 2]. The mandibular condyle has been known to affect the asymmetry of the mandible as an important growth site in the mandible [3, 4]. In addition, mandibular asymmetry has been shown to be influenced by other various factors, such as condylar hyperplasia [5], childhood condylar fractures [6], condylar resorption [7], and internal derangement of the temporomandibular joint (TMJ) [8]. However, most studies [5–8] used two-dimensional radiographs that have several limitations including distortion, magnification, and lack of clarity.

To overcome these limitations, three-dimensional (3D) computed tomography (CT) has been used. Previous studies [9, 10] have shown that the cranial base and the mandible can vary between the deviated and the non-deviated sides. You et al. [11] investigated the mandibular morphology in patients with facial asymmetry and mandibular prognathism using CT data and reported that the length of the mandibular condyle and mandibular body were significantly longer on the non-deviated side than on the deviated side. Oh et al. [12] compared the 3D structure of the mandibular condyles between adults with and without facial asymmetry and reported that menton deviation was associated with right/left differences caused by a smaller condyle, particularly in condylar neck length and neck and head volume, on the deviated side. Moreover, Ikeda et al. [13] found that 3D mandibular morphologic asymmetry was associated with condylar movement in patients with mandibular asymmetry.

In addition, several previous studies have suggested that the shape [14] and volume [14, 15] of the glenoid fossa are necessary to make the diagnosis and administer proper treatment in patients with facial asymmetry. Cho et al. [14] evaluated the effect of the glenoid fossa on menton deviation in facial asymmetry. The vertical position and the depth of the glenoid fossa showed significant differences between the symmetry and asymmetry groups. Kim et al. [15] suggested that the volume of the glenoid fossa, as well as that of the mandibular condyle, should be considered in facial asymmetry.

A literature review of recent CT [11–15] studies on the correlation between 3D morphology of the TMJ and facial asymmetry revealed several measurements that differed significantly in facial asymmetry. However, few studies have simultaneously evaluated the contribution of the mandible and glenoid fossa to menton deviation. The purpose of this study was to identify the factors contributing to menton deviation by evaluating the correlation between menton deviation and 3D morphologies of the mandibular condyle, glenoid fossa, and mandible in facial asymmetry.

## Methods

Ethical approval for this retrospective cohort study was obtained (CNUDH-EXP-2017-016). The sample size of thirty adults was determined using the G*Power 3.1.9.2 software (University of Kiel, Kiel, Germany), with an effect size = 4.92 derived from the preliminary data, *α* = 0.05, and 1-*β* = 0.95. Based on a previous study [12, 14], thirty adults (15 males and 15 females) with skeletal class I or III malocclusion were included. The first selection was performed for subjects with menton deviation on postero-anterior (PA) radiographs exceeding 2° toward the left [16]. Menton deviation was defined as the angle between the vertical reference line drawn from the crista galli to the anterior nasal spine (ANS) and the line drawn from the ANS to the menton on PA radiographs [17, 18]. Finally, the amount of menton deviation was confirmed using CT images. In CT images, menton deviation was defined as the angle between the midsagittal reference plane (MSR plane) and the line drawn from the ANS to the menton on frontal view. The MSR plane was defined as the plane perpendicular to Frankfort horizontal plane (FH plane) passing through the crista galli and opisthion [14]. The right side was the non-deviated side, and the left side was the deviated side because the subjects in this study had menton deviations only toward the left.

The inclusion criteria were subjects over 20 years old with available frontal and lateral cephalograms and CT images acquired before treatment. The exclusion criteria were orthodontic treatment, orthognathic surgery, prosthetic treatment for more than a single crown, TMJ morphological changes, signs or symptoms of temporomandibular disorders, systematic arthritis, facial trauma, craniofacial anomaly, functional discrepancies, functional crossbite, and skeletal class II malocclusion.

The CT images were acquired using a CT scanner (Light Speed QX/i, GE Medical Systems, Milwaukee, WI, USA) before treatment. A more detailed explanation of CT image acquisition can be found in previous studies [12, 14]. The V-works 4.0 software (CyberMed Inc., Seoul, Korea) was used to reconstruct the 3D images from the digital imaging and communication in medicine data. Definitions of the landmarks are described in Table 1 [12, 14]. The 3D reference planes were constructed. The FH plane passed through the right orbitale and right and left porions. The MSR plane was the plane perpendicular to the FH plane passing through the crista galli and opisthion. The anteroposterior reference plane (PO plane) was the plane perpendicular to the FH plane passing through the right and left porions.

**Table 1:** The landmarks used in this study

Landmarks	Abbreviation	Description
Crista galli	Cg	The most superior point of the crista galli of the ethmoid bone
Opisthion	Op	The most posterior point on the posterior margin of the foramen magnum
Porion	Po	The highest point on the roof of the external auditory meatus
Orbitale	Or	The deepest point on the infraorbital margin
Condylion superius	Cd_sup_	The most superior point of the condyle head
Condylion medialis	Cd_med_	The most medial point of the condyle head
Condylion lateralis	Cd_lat_	The most lateral point of the condyle head
Condylion anterius	Cd_ant_	The most anterior point of the condyle head
Condylion posterius	Cd_post_	The most posterior point of the condyle head
Sigmoid notch	S	The most inferior point of sigmoid notch
Gonion lateralis	Go_lat_	The most lateral point of the gonion area
Gonion posterius	Go_post_	The most posterior point of the gonion area
Gonion inferius	Go_inf_	The most inferior point of the gonion area
Antegonion	Ag	The deepest point of antegonial notch of mandible
Menton	Me	The most inferior point on mandibular symphysis
Roof of glenoid fossa	RG	The highest point on the roof of the glenoid fossa
Articular eminence	AE	The most inferior point on the articular tubercle

**Fig. 1:**
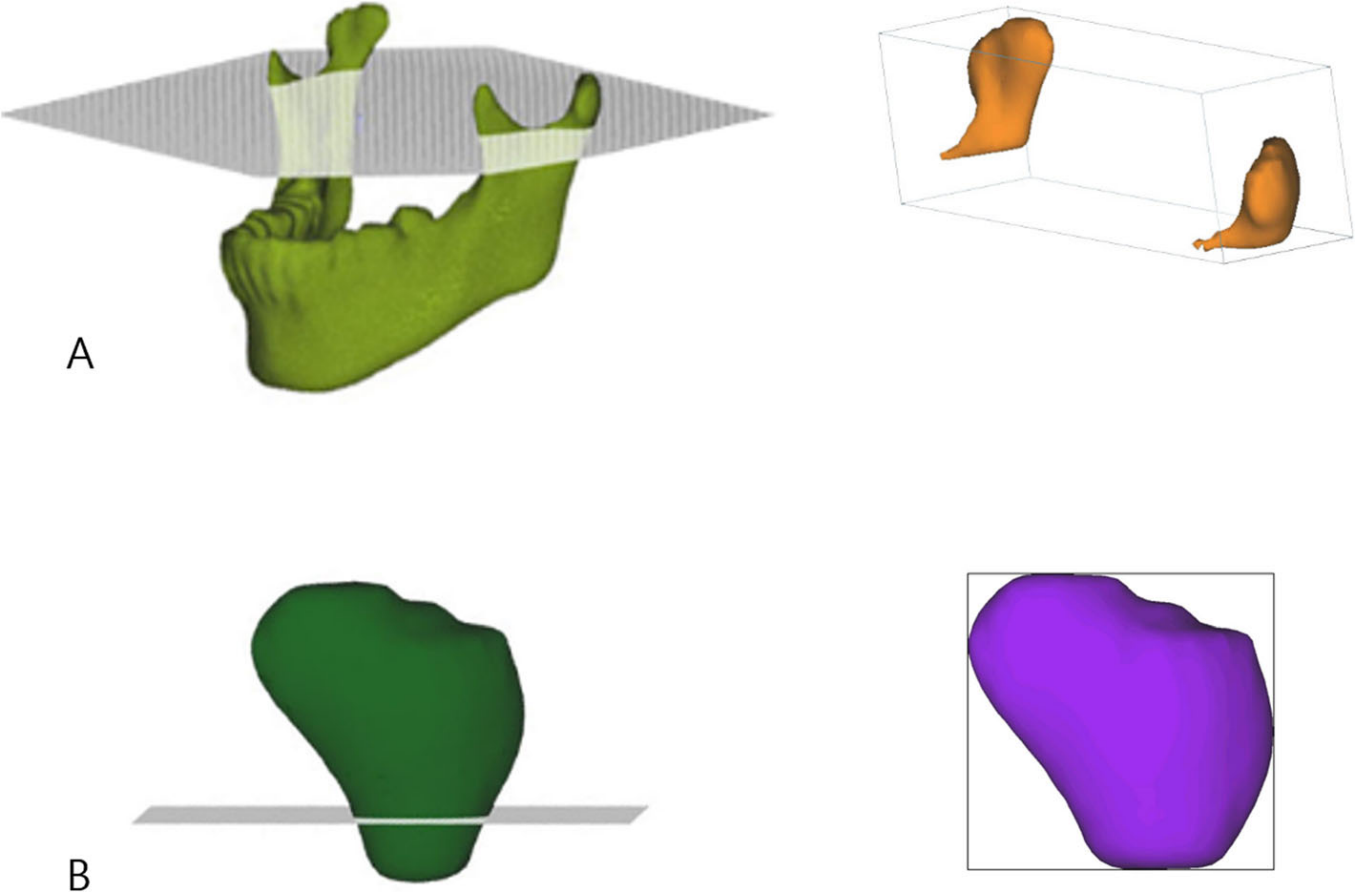
Formation of three-dimensional images. The neck (**a**) and head (**b**) selection of demand (SOD) files were separated from the mandible SOD file that was separated from the whole volume rendering image by removing the overlapping areas using the sculpt functions of the V-works program (CyberMed Inc., Seoul, Korea) (reproduced with permission [12])

In order to precisely identify the landmarks, the mandible was separated from the whole volume rendering image by removing the overlapping areas as reported in a previous study [12] and exporting it into a selection of demand (SOD) file. Moreover, the neck SOD file containing the condylar process above the sigmoid notch was separated by a plane passing through the most inferior point of sigmoid notch while parallel to the FH plane. The head SOD file, which included the upper part of the condylar process, was separated by a plane passing through the most contracted part of the condylar neck while parallel to the FH plane (Fig. 1) [12]. The neck and head SOD files were converted into a 3D surface shaded display (SSD) model to measure the volume of the 3D object model.

**Fig. 2:**
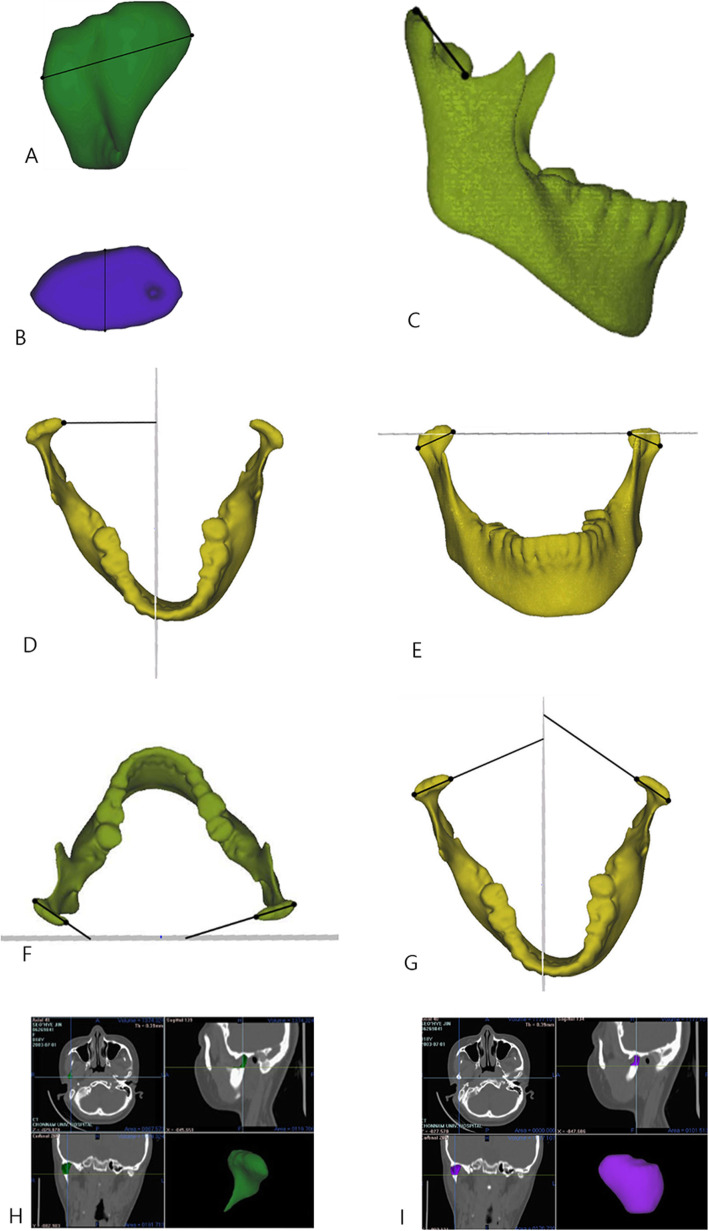
The measurements of the mandibular condyle. **a** Mediolateral dimension of the condyle. **b** Anteroposterior dimension of the condyle. **c** Neck length. **d** Mediolateral condylar position. **e** Condylar angle to the Frankfort horizontal plane. **f** Condylar angle to the anteroposterior reference plane. **g** Condylar angle to the midsagittal reference plane. **h** Neck volume. **i** Head volume (reproduced with permission [12])

**Fig. 3:**
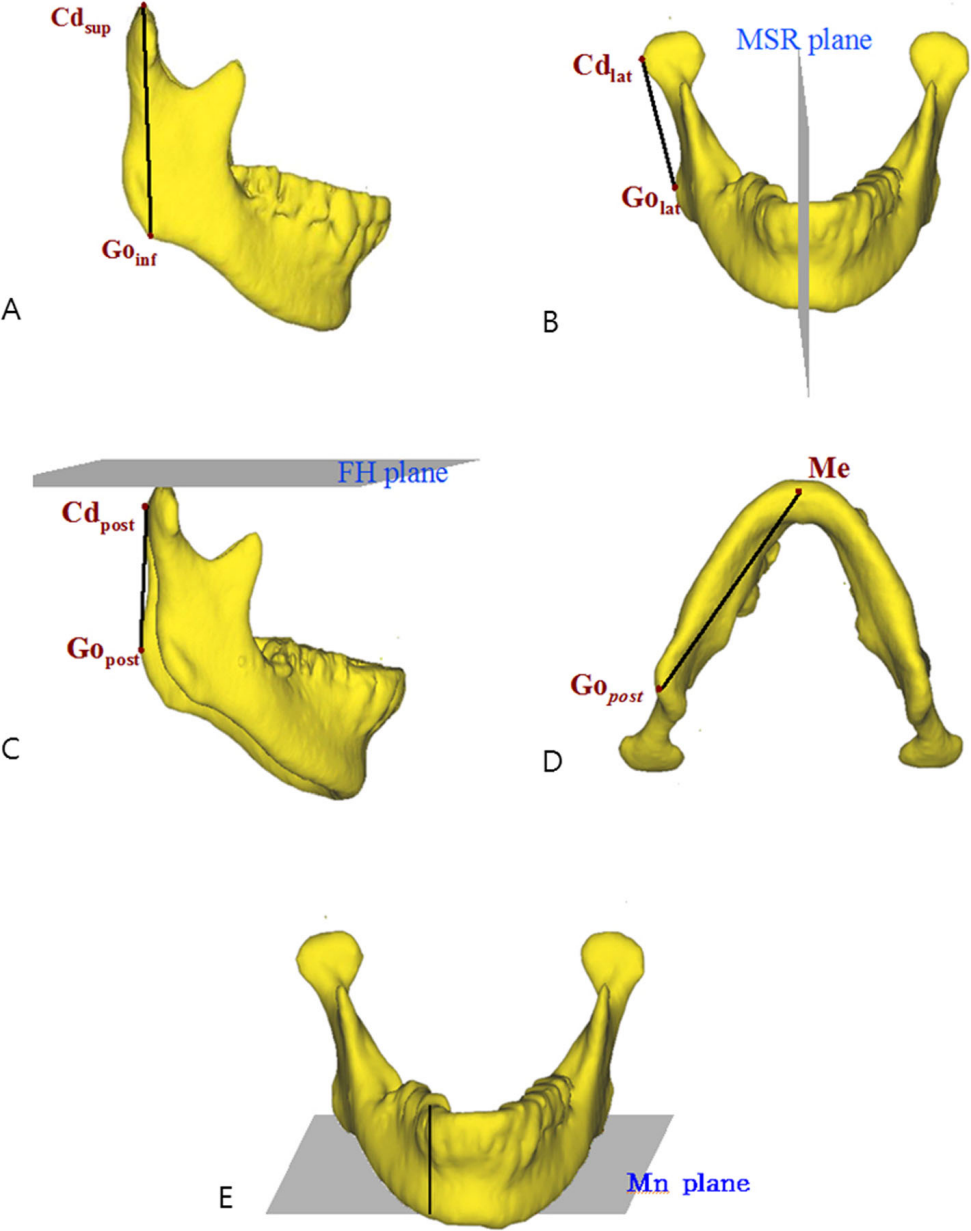
The measurements of the mandible. **a** Ramus length. **b** Frontal ramal inclination. **c** Lateral ramal inclination. **d** Body length. **e** Body height (reproduced with permission [14])

The definition of measurements was described in Table 2 and Figs. 2 and 3 [12, 14]. All measurements, including linear measurements, were measured on a three-dimensional coordinate system, indicating 3D linear, angular, and volumetric measurements. The volumes of the neck and head were calculated automatically using the volume measure function of the V-works program with the SSD model, which is the 3D object model of neck and head.

**Table 2:** Definition of the measurements used in this study

Measurements	Definition
Mandibular condyle	
Mediolateral dimension (mm)	Cd_med_ to Cd_lat_
Anteroposterior dimension (mm)	Cd_ant_ to Cd_post_
Neck length (mm)	Cd_sup_ to *S*
Mediolateral condylar position (mm)	Cd_med_ to MSR plane
Condylar angle to FH (°)	(Cd_med_ - Cd_lat_) to FH plane
Condylar angle to PO (°)	(Cd_med_ - Cd_lat_) to PO plane
Condylar angle to MSR (°)	(Cd_med_ - Cd_lat_) to MSR plane
Neck volume (mm^3^)	The volume of condylar neck above *S*
Head volume (mm^3^)	The volume of condylar head above most constriction point of condylar neck
Glenoid fossa	
Vertical position of RG to FH (mm)	RG to FH plane
Vertical position of AE to FH (mm)	AE to FH plane
Depth of glenoid fossa (mm)	AE to RG parallel to MSR plane
Sagittal position of RG to PO (mm)	RG to PO plane
Sagittal position of AE to PO (mm)	AE to PO plane
Anterior angle of GF to FH (°)	(RG-AE) to FH plane
Mandible	
Ramus length (mm)	Cd_sup_ - Go_inf_
Frontal ramal inclination (°)	(Cd_lat_ - Go_lat_) to MSR plane
Lateral ramal inclination (°)	(Cd_post_ - Go_post_) to FH plane
Body length (mm)	Go_post_ - Me
Body height (mm)	Canine cusp tip to Mandibular plane

*FH* Frankfort horizontal plane, *PO* anteroposterior reference plane, *MSR* midsagittal reference plane, *RG* roof of glenoid fossa, *AE* articular eminence, *GF* glenoid fossa, *Cd*_*med*_ condylion mediais, *Cd*_*lat*_ condylion lateralis, *Cd*_*ant*_ condylion anterius, *Cd*_*post*_ condylion posterius, *Cd*_*sup*_ condylion superius, *S* sigmoid notch, *Go*_*inf*_ gonion inferius, *Go*_*lat*_ gonion lateralis, *Go*_*post*_ gonion posterius, *Me* menton

### Statistical analysis

Statistical analyses were performed using IBM SPSS Statistics (version 23.0; IBM Co., Armonk, NY, USA). All measurements were performed by a single operator (JHC). Twenty images were randomly selected and the measurements were performed twice with a 2-week interval between the measurements. The intraclass correlation coefficient (ICC) was performed to assess reliability. The ICC (> 0.983) indicated excellent intra-observer reliability.

The right/left differences were obtained by subtracting the left value from the right value. Thus, if the value on the deviated side was smaller than that on the non-deviated side, the right/left differences were described as positive (+), otherwise, they were described as negative (−). The results of comparative analysis did not show any statistical difference between the females and males (*P* > .05), so the females and males were combined.

Multiple regression model was used to determine the effect of right/left differences of the measured variables on the menton deviation. A multiple regression model using backward elimination was used to identify the causes of menton deviation. Multiple regression analyses were performed four times for the different conditions: (1) for the mandibular condyle, glenoid fossa, and mandible (*n* = 30); (2) for the mandibular condyle only (*n* = 30); (3) for the glenoid fossa only (*n* = 30); and (4) for the mandible only (*n* = 30).

## Results

The demographic characteristics of the subjects, including gender, age, amount of menton deviation, ANB, and SN-MP are presented in Table 3. The right/left differences of all measurements are shown in Table 4.

**Table 3:** Description of the subjects (*n* = 30)

Demographic characteristic	% or mean ± SD
Gender	
Female (%)	50.0
Male (%)	50.0
Age (years)	23.2 ± 3.8
Amount of menton deviation (°)	5.7 ± 2.5
ANB (°)	−0.5 ± 3.3
SN-MP (°)	34.6 ± 6.3

**Table 4:** The right/left differences of the measurements (*n* = 30)

Measurements	Mean ± SD
Mandibular condyle	
Mediolateral dimension (mm)	0.71±2.19
Anteroposterior dimension (mm)	0.38±1.10
Neck length (mm)	2.43±2.75
Mediolateral condylar position (mm)	0.73±2.08
Condylar angle to FH (°)	2.09±8.13
Condylar angle to PO (°)	−0.59±7.27
Condylar angle to MSR (°)	−3.28±13.42
Neck volume (mm^3^)	341.99±443.68
Head volume (mm^3^)	338.99±379.72
Glenoid Fossa	
Vertical position of RG to FH (mm)	−0.31±2.02
Vertical position of AE to FH (mm)	−0.96±2.45
Depth of glenoid fossa (mm)	−0.66±1.35
Sagittal position of RG to PO (mm)	−0.10±1.51
Sagittal position of AE to PO (mm)	0.21±1.69
Anterior angle of GF to FH (°)	−3.37±7.34
Mandible	
Ramus length (mm)	3.27±4.22
Frontal ramal inclination (°)	3.98±4.53
Lateral ramal inclination (°)	3.25±5.42
Body length (mm)	1.16±3.32
Body height (mm)	0.19±1.42

*SD* standard deviation, *FH* Frankfort horizontal plane, *PO* anteroposterior reference plane, *MSR* midsagittal reference plane, *RG* roof of glenoid fossa, *AE* articular eminence, *GF* glenoid fossa

The regression equation between menton deviation and the right/left differences of the measurements for all 20 variables (*n* = 30) was as follows: menton = 0.735 • (neck length) + 0.329 • (ramus length) + 0.331 • (frontal ramal inclination). The adjusted *R*^2^ value was 0.765 (*P* < .000). The regression equation between menton deviation and the right/left differences of the nine variables for the mandibular condyle (*n* = 30) was as follows: menton = 0.903 • (neck length) + 0.005 • (head volume). The adjusted *R*^2^ value was 0.699 (*P* < .000). The regression equation for the six variables of the glenoid fossa (*n* = 30) was as follows: menton = −1.022 • (vertical position of AE to FH). This equation showed that the adjusted *R*^2^ value was 0.157, indicating that the glenoid fossa had almost no effect on menton deviation (*P* < .05). The regression equation for the five variables of the mandible (*n* = 30) was as follows: menton = 0.659 • (ramus length) + 0.407 • (frontal ramal inclination). In this equation, the adjusted *R*^2^ value was 0.686 (*P* < .000, Table 5).

**Table 5:** Multiple linear regression analysis between the menton deviation and the right/left differences of the measurements (*n* = 30)

Measurements	Coefficient	*t*	*P* value	VIF
For all variables^a^				
Neck length	0.735	3.223	0.003**	2.315
Ramus length	0.329	2.082	0.017*	2.346
Frontal ramal inclination	0.331	3.031	0.005**	1.430
For the mandibular condyle^b^				
Neck length	0.903	3.698	0.000***	2.072
Head volume	0.005	2.625	0.014*	2.072
For the glenoid fossa^c^				
Vertical position of AE to FH	−1.022	2.567	0.016*	NA
For the mandible^d^				
Ramus length	0.659	4.733	0.000***	1.363
Frontal ramal inclination	0.407	3.305	0.003**	1.363

*VIF* variance inflation factor, *AE* articular eminence, *FH* Frankfort horizontal plane, *NA* not available

^a^Adjusted *R*^2^ = 0.765, *P* < 0.000

^b^Adjusted *R*^2^ = 0.699, *P* < 0.000

^c^Adjusted *R*^2^ = 0.157, *P* < 0.05

^d^Adjusted *R*^2^ = 0.686, *P* < 0.000

^*^P < 0.05

^**^P < 0.01

^***^P < 0.001

## Discussion

The degree of recognition of facial asymmetry can be affected by various factors. Ahn et al. [18] reported that the degree of asymmetry recognition increased when the degree of menton deviation increased. Lee et al. [17] also reported that menton deviation had the greatest effect on the degree of facial asymmetry recognition. In addition, Ferguson [16] evaluated the correlations between facial photographs and PA radiographs of patients with facial asymmetry and reported that the asymmetry was recognized when menton deviation from the midline was 2° or higher. McAvinchey et al. [19] investigated the perception of facial asymmetry in young adults and reported that the perception of asymmetry was affected by the amount of asymmetry. Menton deviation might be one of the primary contributing factors for recognition of facial asymmetry. Thus, it is necessary to evaluate the factors contributing to menton deviation in order to establish treatment plans for facial asymmetry. The present study examined the factors contributing to menton deviation in order to determine what contributed to facial asymmetry.

The subjects had skeletal class I or III malocclusion, in which the ANB angle was lower than 5° on the lateral cephalogram. Ngan et al. [20] compared the skeletal growth changes between class II division 1 and class I subjects and reported that the majority of the class II cases showed mandibular skeletal retrusion or a combination of horizontal and vertical abnormalities of the mandible, rather than maxillary protrusion. Previous studies [3, 21] compared the position and volume of the condyle in class I, II, and III and reported that the greatest condylar decentralization was observed in the class II group [21] and significantly lower condylar volume was observed in class II subjects compared to those in class I and III [3]. Thus, the present study excluded subjects with skeletal class II malocclusion due to the possible presence of mandibular growth disorders [3, 20, 21].

In this study, all measurements, including volumes of condylar neck and head, showed no significant differences between males and females. These results are inconsistent with the results of previous studies [3, 4]. Previous studies [3, 4] reported that the condylar volume was significantly higher in males than in females. This conflicting result is thought to be due to the fact that the previous studies [3, 4] was conducted on a sample size of 200 and 94, respectively, while the present study was conducted on only 30 subjects.

**Fig. 4:**
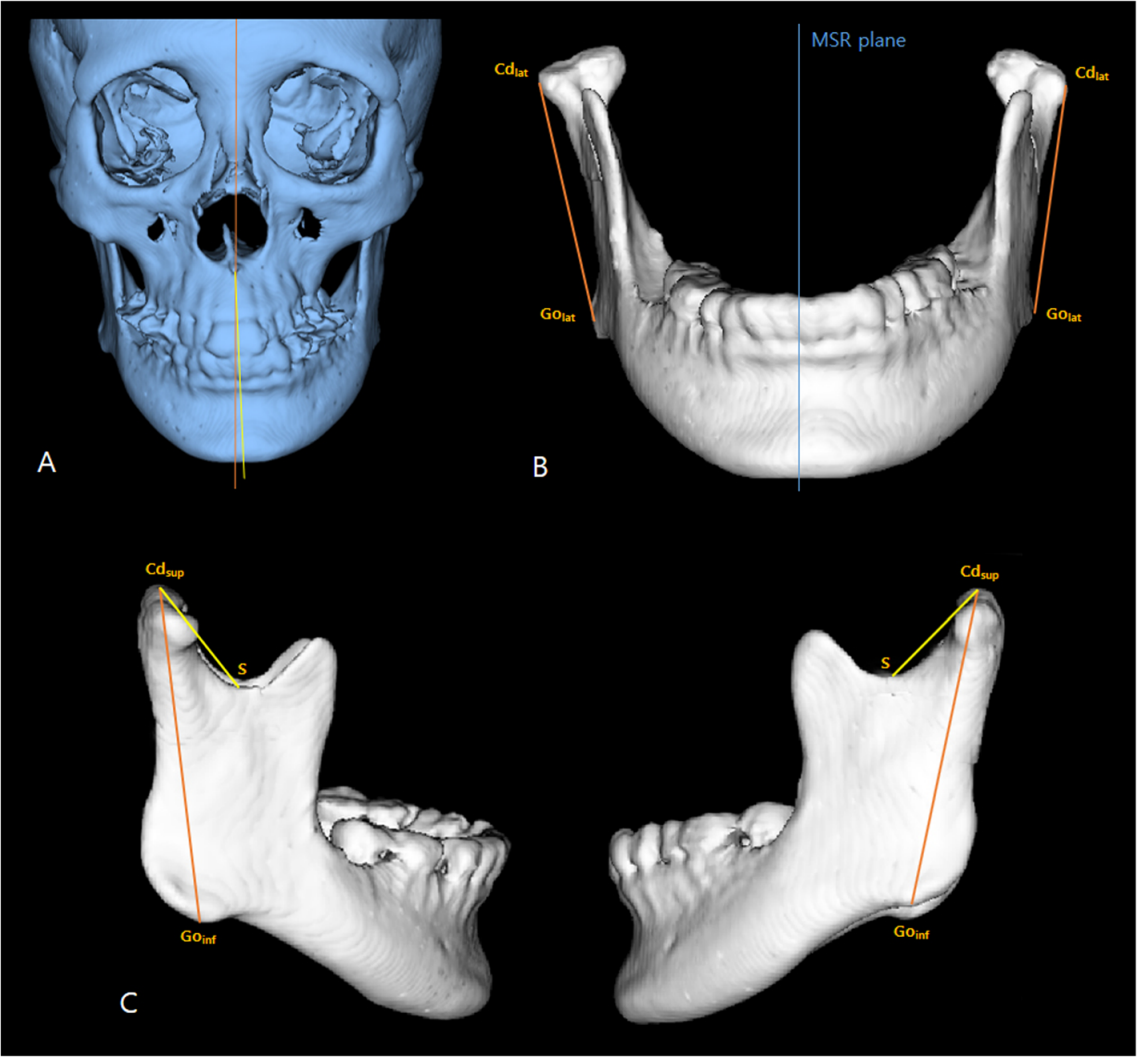
A subject consistent with the results of this present study. **a** Menton is deviated to the left side. **b** The frontal ramal inclination is greater in the right side (non-deviated side) than the left side (deviated side). **c** The ramus length and neck length are greater in the right side (non-deviated side) than the left side (deviated side)

In facial asymmetry, the explanatory power of the regression equation for all 20 variables for the mandibular condyle, glenoid fossa, and mandible was 76.5%, and the neck length, ramus length, and frontal ramal inclination had positive influences on menton deviation. Multiple regression analysis of the anatomical structures affecting menton deviation showed that the explanatory power of the regression equation for the mandibular condyle was 69.9% and the neck length and head volume of the mandibular condyle had positive influences on menton deviation. The explanatory power of the regression equation for the glenoid fossa was 15.7%, indicating that the glenoid fossa had little effect on menton deviation. The explanatory power of the regression equation for the mandible was 68.6% and the ramus length and frontal ramal inclination had positive influences on menton deviation (Table 5). The variance inflation factor for all variables was less than 3, indicating that there was no multicollinearity. These results suggest that the right/left differences of the mandibular condyle and mandible can be used to predict menton deviation. Specifically, the right/left differences of neck length and ramus length contributed the most to menton deviation. However, it was not possible to predict menton deviation using the right/left difference of the glenoid fossa (Fig. 4). These results are caused by the mandibular asymmetry is associated with the condylar growth center, which directly or indirectly regulates the size of the condyle and also the length of the condylar neck, ramus, and body of the mandible [22]. In the diagnosis of facial asymmetry patients, the difference between right and left side of mandible including mandibular condyle should be considered and a treatment plan should be established to improve it.

The present study had several limitations. Although the upper and middle thirds of the face have less effect on facial asymmetry than the lower third of the face [17, 19], there might be a maxillary asymmetry in facial asymmetry. The horizontal reference plane, FH plane, was defined as a plane passing through the right orbitale and right and left porions to exclude the asymmetry on orbitale points. Nevertheless, the effects of the maxillary asymmetry could not be completely ruled out, due to the use of ANS for measuring the menton deviation.

In addition, menton deviation can be influenced by other factors, such as functional adaptation and the surrounding neuromuscular system. Maki et al. [23] evaluated the correlations between the surrounding muscles and bone density in an asymmetrical mandible and reported that the asymmetrical mandible was associated with asymmetrical distributions of the highest mineralized cortical bone and that it was age dependent. Nakano et al. [24] evaluated changes in the calcified tissue of the mandibular condyle during altered muscle function and showed that both the mandible and the condyle modified their shape and size, as well as the trabecular bone of the condyle, during the shifting of the mandible to one side as it closed. Nur et al. [25] found that soft tissues compensated for hard tissues at the gonial level. In addition, Kurusu et al. [26] evaluated the relationship between occlusal force and mandibular condyle morphology and reported that occlusal force influenced not only maxillofacial morphology but also mandibular condyle morphology. Thus, further studies are needed to evaluate the effects of soft tissue and function to better understand the etiology of facial asymmetry.

In the literature, the condylar volume has been also related to the type of mandibular divergence [27]. In a study with ninety-four subjects, higher condylar volume was a common characteristic of low angle subjects compared to normal and high mandibular plane angle subjects. In this study, the differences according to the type of mandibular divergence were not evaluated. Thus, the role of the neck and ramus length related to the concept of mandibular divergence should be supported by new studies.

## Conclusions

In facial asymmetry, the right/left differences in mandibular condyle and mandible have more impact on the menton deviation than the right/left differences in the glenoid fossa.

## Data Availability

Not applicable
